# The Impact of Pediatric Palliative Care Education on Medical Students' Knowledge and Attitudes

**DOI:** 10.1155/2013/498082

**Published:** 2013-12-31

**Authors:** Aleksandra Korzeniewska-Eksterowicz, Łukasz Przysło, Bogna Kędzierska, Małgorzata Stolarska, Wojciech Młynarski

**Affiliations:** ^1^Pediatric Palliative Care Unit, Department of Pediatrics, Oncology, Hematology and Diabetology, Medical University of Lodz, 36/50 Sporna Street, 91-738 Łodz, Poland; ^2^Gajusz Foundation, Pediatric Palliative Care Center, Home Hospice for Children of Lodz Region, 87 Dąbrowskiego Street, 93-271 Lodz, Poland; ^3^Institute of Psychology, University of Lodz, 10/12 Smugowa Street, 91-433 Lodz, Poland; ^4^Department of Pediatrics, Oncology, Hematology and Diabetology, Medical University of Lodz, 36/50 Sporna Street, 91-738 Łodz, Poland

## Abstract

*Purpose*. Most undergraduate palliative care curricula omit pediatric palliative care (PPC) issues. Aim of the study was to evaluate the pilot education programme. *Methods*. All 391 students of Faculty of Medicine (FM) and 59 students of Division of Nursing (DN) were included in anonymous questionnaire study. Respondents were tested on their knowledge and attitude towards PPC issues before and at the end of the programme and were expected to evaluate the programme at the end. *Results*. For final analysis, authors qualified 375 double forms filled in correctly (320 FM and 55 DN). Before the programme, students' knowledge assessed on 0–100-point scale was low (FM: median: 43.35 points; 25%–75%: (40p–53.3p); DN: 26.7p; 13.3p–46.7p), and, in addition, there were differences (*P* < 0.001) between both faculties. Upon completion of the programme, significant increase of the level of knowledge in both faculties was noted (FM: 80p; 73.3–100; DN: 80p; 66.7p–80p). Participation in the programme changed declared attitudes towards some aspects of withholding of special procedures, euthanasia, and abortion. Both groups of students positively evaluated the programme. *Conclusions*. This study identifies medical students' limited knowledge of PPC. Educational intervention changes students' attitudes to the specific end-of-life issues. There is a need for palliative care curricula evaluation.

## 1. Introduction

In the majority of Polish medical universities, palliative medicine is a part of the teaching programme [1]. However, most programmes in Poland and worldwide include only adult palliative care [1–8]. Gibbins et al. [6] suggest that 86% of medical curriculum omits PPC. There are scarce literature reports on teaching programmes within pediatric palliative care (PPC) [9–11]. In Poland, PPC has been mainly provided by specialized pediatric palliative home care teams, for example, home hospices [12–14]. Currently, there are almost 40 pediatric home hospices and, in the years 2000–2010, the number of treated children had increased more than fivefold [14]. Despite dynamic development of the PPC, there are difficulties in staff recruitment and significant deficits in the knowledge about PPC. These difficulties arise as a result of a lack of PPC training among medical students.

Pediatric Palliative Care Unit is the first academic unit in Poland which implemented teaching of PPC and developed the first pilot training programme in the field of PPC in Poland.

PPC became a part of the general obligatory pediatrics curriculum. It applies to final year medical students (Faculty of Medicine, FM) and 3rd year nursing students (Division of Nursing, DN). The module for FM consists of two days of compulsory lectures (5 hours) and workshops (5 hours). The module for DN consists of 5 hours of compulsory lectures. During the first day, students are divided into groups of 10–12 people. The second day (only for FM students) takes a form of workshops (in groups of 5-6 students) divided into two parts: clinical and psychological. Clinical workshops focus on detailed case studies of hospice patients. The students are given written materials with 18 case studies. Due to the fact that the patients stay at home, where the hospice team visits them, their active participation in the training was logistically not possible.

Aim of the study was to examine whether the programme will have a significant effect upon students' knowledge and attitude towards PPC issues and to evaluate the feedback of students who participated in the education programme.

## 2. Material and Methods

All students participating in the programme were included in our study (391 FM students and 59 DN students).

### 2.1. Evaluation of Students' Knowledge and Attitude

We conducted an anonymous questionnaire study. Respondents filled in identical forms before and after completion of the programme. The questionnaire covered selected social and demographic data and 28 closed questions evaluating the knowledge and attitude of students towards the PPC. Nine questions concerned scientific PPC issues, and the remaining questions concerned attitudes of respondents towards selected aspects of PPC, including their attitude towards special procedures, euthanasia, and abortion.

In 9 questions evaluating the knowledge of students, each correct answer scored 1 point, which gave 15 points maximum (some questions were multichoice questions). During analysis, the results were standardized by transforming the raw results onto a 100-grade scale (0–100 points).

### 2.2. Evaluation of Students' Satisfaction from the Programme

The participants were expected to evaluate the education programme in the form of an anonymous questionnaire given to them at the end of the programme. The questionnaire designed for the purpose of that research consisted of 10 closed and 4 open questions. Closed questions covered the following issues: form and structure of the programme, lectures and workshop evaluation, content evaluation, usefulness of subject and its relevancy to the practical and academic work, and assessment of the tutors and the conspectus given to the students. The students were expected to assess each aspect of the programme on a 0–6-point scale, where 0 indicated complete lack of satisfaction and 6 complete satisfaction. The open questions gave students a chance to freely express their opinions and reflections as well as suggest which aspects of the programme were unnecessary or missing. Student's evaluation was voluntary and did not affect their assessment.

#### 2.2.1. Statistical Analysis

Continuous variables of paired observations were performed by Wilcoxon test or *t*-test depending on variable distribution. Post hoc comparisons were performed with Tukey's HSD test if statistical significance was noted in analysis of variance (ANOVA). Noncontinuous variables were studied using chi-square test with corrections for multiple comparisons. A *P* value <0.05 was considered as statistically significant. STATISTICA 10.0 (Statsoft, Tulsa, OK, USA) software was used for statistical analysis. The study was approved by the Ethics Committee of Medical University of Lodz (RNN/136/10/KE).

## 3. Results

### 3.1. Evaluation of Knowledge and Attitudes of Students

All students participating in the programme completed the questionnaires; for final analysis, we qualified 375 double forms filled in correctly (320 FM and 55 DN). Social and demographic characteristics of respondents are presented in Table 1.

**Figure 1:**
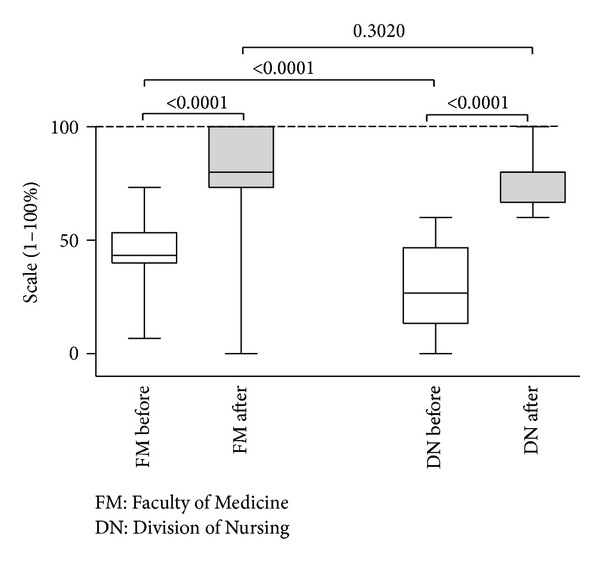
The level of knowledge of Faculty of Medicine students (left panel) and Division of Nursing students (right panel) before and after completion of the obligatory programme.

Before the programme, the knowledge of students from both faculties on PPC was low (FM: median: 43.35 points (p), 25%–75%: (40p–53.3p); DN: median: 26.7p, 25%–75%: (13.3p–46.7p)), and, in addition, there were statistically significant (*P* < 0.001) differences between both faculties. Upon completion of the programme, statistically significant increase of the level of knowledge in both faculties was noted (FM: median: 80p, 25%–75%: 73.3–100; DN: median: 80, 25%–75%: 66.7–80), as shown in Figure 1.

Before the programme, both groups of students had great problems with questions concerning the structure of patients referred for palliative care and criteria of morphine use in children. Only 6% of FM students knew that the largest group of hospice care children constitute those with neurological diseases, and none from DN students gave proper answer. The most common choice was oncological disease (81% versus 65%). After the programme, the majority of students *P* < 0.001 gave correct answer (94% versus 91%). Before the programme, 24.4% of FM and 43.6% of DN students thought that morphine is contraindicated in treatment of newborns. After the programme, the majority of students (97.5% versus 100%) gave correct answer (*P* < 0.001). Before education, only 7.8% of FM and 9.1% of DN students knew inhaled way of morphine application and after the programme, most of them (74.6% versus 72.7%) gave proper answer (*P* < 0.001). Additionally, up to 48.1% of FM and 65.5% of DN students indicated respiratory depression as most common complication of treatment; after the education, that answer was indicated only by 7.2% of FM and 7.3% of DN students.

**Figure 2:**
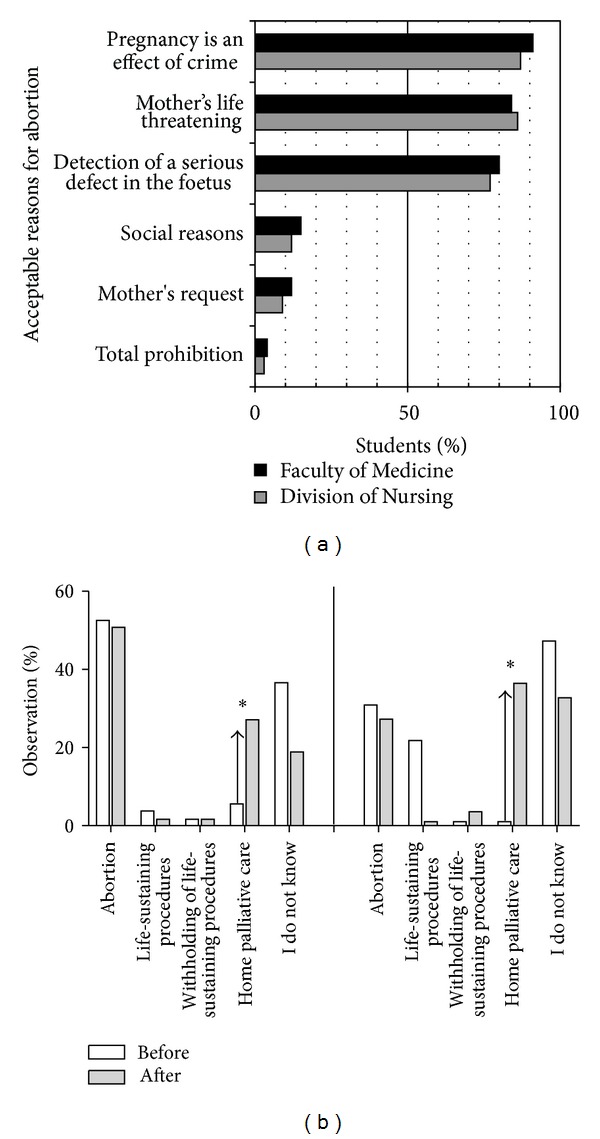
Students' opinion on specific problems of pediatric palliative care. (a) Percentage of respondents acceptable reasons for abortion. (b) The percentage of Faculty of Medicine students' (left panel) and Division of Nursing (right panel) students' declaring choice of particular management if a lethal defect was detected in their child. *P < 0.05.

The questionnaire included students' opinion on specific problems of PPC, including withholding of special procedures, euthanasia, and abortion, Table 2 and Figure 2. The students listed acceptable, according to them, reasons for abortion. There were no differences between the students' groups and participation in the programme did not change their declared attitudes, Figure 2(a). Students were asked what they would do if a lethal defect would be detected in their child during prenatal diagnostics, Figure 2(b).

### 3.2.  Evaluation of Students' Satisfaction from the Programme

The questionnaires were filled in by all participants; eventually, 383 properly filled forms were analyzed (336 FM and 47 DN).

Both groups of students positively evaluated the programme. Mean overall assessment was 4.85 (FM) and 5.02 (DN) points; form of the programme assessment was, respectively, 4.87 and 4.83, structure of the programme was 5.03 and 4.97, conspectus given to the students was 5.68 and 5.85, lectures were 4.87 and 4.92, and workshop evaluation was 4.79 (FM only).

**Figure 3:**
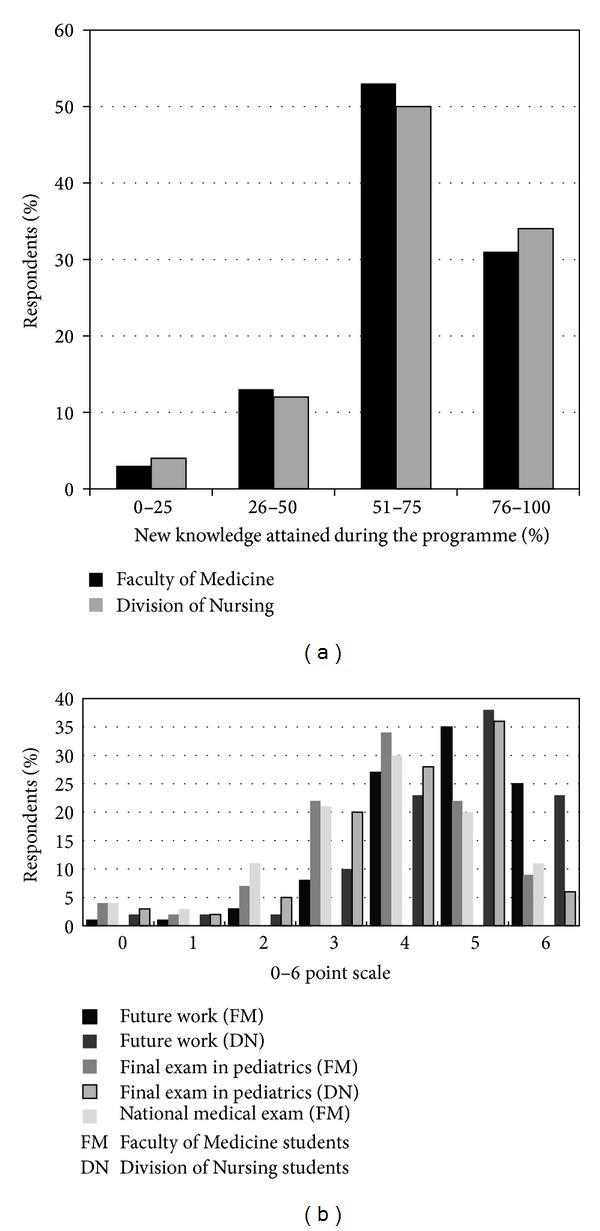
Students' opinion on knowledge attained during the programme. (a) Evaluation of novelty of knowledge attained during the programme. (b) The usefulness of the new knowledge in students' future work and preparation for exams evaluated on 0–6-point scale.

For the majority of participants, the knowledge obtained during the programme was new, Figure 3(a). The respondents were asked how useful the new knowledge will be in their future work and preparation for exams; they evaluated the usefulness on 0–6-point scale. The majority of students positively answered this question (5 and 6 points), Figure 3(b). The students unequivocally (95.2% FM and 95.7% DN) expressed the need to introduce palliative care of children into the pediatric curriculum during studies.

All the lectures and teachers were evaluated individually by students. All teachers had similar favorable ratings; their work was well received and graded on average of 5.26 ± 0.52. Additionally, individual evaluation of each teacher's domain showed mean results above 5 points.

Answering the opened questions, only 12 students expressed their negative opinion about the programme and suggested that the subjects were unnecessary. The students were given a chance to suggest the elements of the programme which needed modification. Most of students (80.1% FM and 85.1% DN) said that the programme should be extended in terms of the amount of teaching hours; 66.9% FM of students wanted more workshop hours and 86.9% suggested a strong need for more psychological workshops. Additionally, 72% of FM and 82.9% of DN students expressed a need for patient's participation in the teaching.

## 4. Discussion

The authors of many reports stress the need of pre- and postgraduate training in palliative care [1–8]. Unfortunately very few studies have been devoted to education in PPC-discipline which is very distinct from palliative care of adults [9–11, 15]. The specific aspects of PPC lead to the fact that, in Poland, PPC is carried out mainly by pediatricians and only in special cases by specialists in palliative medicine. Thus, naturally, PPC was integrated with pediatric curriculum. Low initial level of knowledge on PPC issues among students from both faculties and its significant improvement after classes are strong argument for the introduction of such classes into curriculum. The results are similar to observations from other authors, which confirm efficacy of such educational intervention [2–8, 16–18]. The observed lower initial level of knowledge among DN students may result from the fact that the programme was carried out at the 3rd year of studies, and not the 6th year as it was the case for MF students. The profile of questions which were the most troublesome for students also confirms the need to incorporate PPC issues in the curriculum. The most difficult questions concerned basic clinical issues and this is a valuable comment for the authors of this research, especially in the light of our previous experience of providing PPC. In the last few years of providing hospice care, we have observed some lack of knowledge among the medical staff in terms of home based care and the rules of admitting patients. Very often, the referred children did not require palliative care but a long-term treatment, in cases such as cerebral palsy or encephalopathy. The problem of pain management is included in all PPC training programmes, and the participants almost always stress how insufficient their knowledge on this issue is [10, 19–21]. The observed differences in results between faculties may result from differences in teaching curricula.

Ethical and moral considerations in end-of-life care are an important element of PPC education [17, 22–25]. During workshops and lectures, issues specific for perinatal palliative care and euthanasia were discussed. The problem of students' attitude towards euthanasia has been a subject of many studies [26–32], but, in the available Polish literature, there are only a few reports [33–37]. The results of Polish studies published by Leppert et al. in 2005 and 2009 demonstrated that 82% and 58% of medical students, respectively, definitely rejected possibility of performing euthanasia act, while 6% and 29%, respectively, were indecisive [33]. The questionnaires were distributed after classes on palliative care or bioethics. In the study by Mierzecki et al., conducted among 1st year students, almost 50% declared acceptance of euthanasia [34]. Similar results were obtained by Reczek [35] and Erderle et al. [36]. The analysis of other authors' results indicates that students of higher years are less accepting towards euthanasia [28]. The results of our study only partially seem to confirm the above observation. Many authors claim that participation in classes on palliative care may also be the reason, but we did not analyze whether these students participated in classes on palliative care for adults before. The obtained results differ from those reported by some authors; however, influence of religious beliefs as the element of cultural differences among countries is always stressed. This may be the reason why in USA, The Netherlands, Greece, and Hungary most of the students supported euthanasia and physician-assisted suicide (PAS), but, in countries such as Norway, Sweden, Yugoslavia, Italy, Germany, Sudan, and Malaysia, most students expressed negative positions regarding euthanasia [38]. In the presented study, students' opinions on the introduction of legal regulations concerning euthanasia in Poland are interesting. More than 70% of DN students did not see possibility of euthanasia in their patients and were against the legalization of euthanasia, while 88% of FM students would not perform euthanasia act, but, at the same time, almost 60% support legalization of euthanasia if certain criteria are met. Euthanasia is illegal in Poland; thus, the above considerations are purely theoretical. However, we may claim that these attitudes result from, on the one hand, respect for patient's autonomy and his right to decide about his own life and, on the other hand, from granting the doctor, right to refuse performing a procedure against his philosophy of life (the so-called conscience clause, acceptable in the Polish law). In addition, it is noteworthy that, after the programme, the number of students in both faculties, who were opponents of legalization of euthanasia and PAS, significantly increased. These results are in contrast to those of Leppert, where supporters of euthanasia were by half less numerous [33]. It is possible that these differences resulted from the fact that answers to that question included also an option of euthanasia only after certain criteria have been fulfilled. In the study, general attitude towards euthanasia was confronted with hypothetical situation of individual management choice in perspective of an untreatable disease. Before classes, almost 40% of responders declared euthanasia or PAS in such case, but participation in the programme decreased significantly the number of persons declaring choice of the above options. Interpretation of the obtained results leads us to conclusions that discussions with students changed their stereotypic attitudes and helped to overcome individual fear of suffering and death, resulting from lack of knowledge. The observed change in the students' attitude may be regarded as an added value of the implemented educational programme, which is consistent with current tendencies in the preparation of curriculum for medical students.

As a result of the advancements in the prenatal methods of diagnosis, there is a relatively new area of PPC: perinatal palliative care. It applies to situations in which prenatal diagnosis suggests serious, untreatable pathology of the foetus. The current literature suggests that PPC programmes may be comprehensive, integrative and initiated early already during pregnancy [38, 39]. Elements of perinatal palliative care have been introduced into the presented programme and during workshops issues connected with initiating special procedures or abortion, in particular in a situation of unfavorable prenatal diagnosis, were discussed. The majority of respondents accepted possibility of refraining from resuscitation in case of untreatable disease in a child, and these results are not surprising in the context of current legal regulations in Poland. Additionally, these problems are discussed during classes of pediatrics and ethics. Certain issues connected with abortion require additional discussion. The Polish law accepts abortion in 3 situations: (1) detection of a serious defect in the foetus, (2) if mother's life is threatened, or (3) pregnancy is an effect of crime. The majority of respondents accepted the above conditions. In the majority of reports, regardless of country of origin, there is consensus on abortion acceptance if mother's life is threatened, but the greatest differences, resulting probably from social and religious beliefs, concern abortion on mother's request [40–44]. Similar to the problem of euthanasia, general attitude of responders to abortion was confronted with hypothetical necessity of decision making in a situation of prenatal diagnosis of a lethal defect in responder's own child. It seems that, after the programme, students perceive palliative care as an alternative to management showing features of aggressive persistent treatment for families who reject possibility of abortion. These observations are invaluable as they reflect the very ideas, aims, and principles of palliative care [38, 39].

The above presented educational programme was the first pilot initiative in the country and that is why student evaluation of the project is so crucial for the tutors. The respondents highly valued the programme. The presented results are similar to observations of other authors. Schiffman et al. [11] and Kato et al. [10] demonstrated in their studies that participants of PPC training positively evaluated the programme and its applicability. In the research conducted by Michelson et al. [45], residents reported mainly no or minimal training, knowledge, experience, comfort, and competence in palliative care communication and symptom management. Michelson et al. [45], Billings and Block [46] showed a preference for education via observation and participation in multidisciplinary groups.

In Poland, palliative care for children has been mainly provided by specialized pediatric palliative home care teams. Due to the logistics (the distance between the hospice's office and the children's homes varies from 15 to 100 kilometres) there was no possibility of bedside teaching; however, there is also a plan to open a hospice ward for children within the next two years. In the presented research, the students expressed a need for more teaching in psychology. Those observations confirm the results of other research. Previous studies have showed that palliative medicine is a rich area to provide teaching about other forms of medical practice to students such as the patient-doctor communication, relationship, caring, and empathy [9, 47–51].

There are some limitations to this study that should be kept in mind when interpreting the findings. Evaluation of the efficacy of training and changes in the students' attitudes was performed directly after completion of the programme. Thus, long-term influence of training on the level of knowledge and attitudes of respondents is difficult to determine. In future, a follow-up study should be considered, in order to properly evaluate the effects of training; however, performing of such a follow-up study may be difficult from the logistic point of view. The authors may have difficulties with distribution of the questionnaire among absolvents, who will have started working in various centres around Poland by then, and the university does not collect such data. Additionally, it should be remembered that the programme had a pilot character and the presented results should be considered as initial, serving as a starting point for future research.

The results presented indicate that the outcome of pilot paediatric palliative care educational programme was really feasible and well received by the students. Pregraduate educational intervention may improve cooperation of home hospice team with GPs and hospitals staff. However, the number of hours devoted to pediatric palliative care in the curriculum is short and some issues, especially psychological, are not covered enough.

## Figures and Tables

**Table 1:** Social and demographic characteristics of respondents.

	Faculty of Medicine students, *n* = 320		Division of Nursing students, *n* = 55	
	*n*	%	*n*	%
Gender				
Female	105	32.8%	0	0%
Male	215	67.2%	55	100%
Place of living				
Rural	39	12.5%	7	12.7%
Small town (<100.000 inhabitants)	66	21.2%	12	21.8%
Town (100–500.000)	37	11.9%	7	12.7%
Big city (>500.000)	170	54.5%	29	52.7%
Having children				
Yes	265	83.1%	48	87.3%
No	54	16.9%	7	12.7%
Parents' education level				
Primary	4	1.3%	12	21.8%
Secondary	105	34.0%	24	43.6%
Academic	200	64.7%	19	34.5%
Medical profession in close family				
Physician	105	32.8%	0	0%
Nurse	14	4.4%	10	18.2%
Pharmacist	11	3.4%	0	0%
Others	22	6.9%	0	0%
Religion				
Catholic	278	87.1%	48	87.3%
Protestant	1	0.3%	0	0%
Orthodox	0	0%	0	0%
Muslim	0	0%	0	0%
Atheist	35	11%	7	12.7%
Others	5	1.6%	0	0%

**Table 2:** Students' opinion on specific problems of palliative care before and after education programme.

Question and answer's option	Percentlty of Medicine students		P value* within the group	Percentage of indicated answers by Division of Nursing students		*P* value* within the group	*P* value* between the groups	
	Before	After		Before	After		Before	After
Are there situations when resuscitation of a child may not be initiated?								
Yes	91.3	98.1	0.001	56.4	100	<0.001	<0.001	0.588
No	2.2	0.6		25.5	0			
I do not know	6.6	1.3		18.2	0			
Would you decide on euthanasia act in a patient?								
Yes	11.5	9.5	0.419	21.8	9.1	0.065	0.035	0.925
No	88.5	90.5		78.2	90.9			
What would you choose in situation of suffering from a progressing, untreatable disease?								
Natural death	59.7	75.4	<0.001	87.3	76.4	0.138	<0.001	0.1
Euthanasia	28.4	17.9		12.7	23.6			
Physician-assisted suicide	11.8	6.7		0	0			
Should be euthanasia legally available in Poland?								
Yes	2.5	3.5	0.048	0	3.6	0.009	<0.001	0.895
Yes, but only if special criteria had been fulfilled	58.5	47.9		28	52.7			
No	39	48.6		72	43.6			

*Pearson's *χ* ^2^Test.
